# Nencki Genomics Database—Ensembl funcgen enhanced with intersections, user data and genome-wide TFBS motifs

**DOI:** 10.1093/database/bat069

**Published:** 2013-10-01

**Authors:** Izabella Krystkowiak, Jakub Lenart, Konrad Debski, Piotr Kuterba, Michal Petas, Bozena Kaminska, Michal Dabrowski

**Affiliations:** Laboratory of Molecular Neurobiology, Neurobiology Center, Nencki Institute of Experimental Biology, Warsaw, Poland

## Abstract

We present the Nencki Genomics Database, which extends the functionality of Ensembl Regulatory Build (funcgen) for the three species: human, mouse and rat. The key enhancements over Ensembl funcgen include the following: (i) a user can add private data, analyze them alongside the public data and manage access rights; (ii) inside the database, we provide efficient procedures for computing intersections between regulatory features and for mapping them to the genes. To Ensembl funcgen-derived data, which include data from ENCODE, we add information on conserved non-coding (putative regulatory) sequences, and on genome-wide occurrence of transcription factor binding site motifs from the current versions of two major motif libraries, namely, Jaspar and Transfac. The intersections and mapping to the genes are pre-computed for the public data, and the result of any procedure run on the data added by the users is stored back into the database, thus incrementally increasing the body of pre-computed data. As the Ensembl funcgen schema for the rat is currently not populated, our database is the first database of regulatory features for this frequently used laboratory animal. The database is accessible without registration using the mysql client: mysql –h database.nencki-genomics.org –u public. Registration is required only to add or access private data. A WSDL webservice provides access to the database from any SOAP client, including the Taverna Workbench with a graphical user interface.

**Database URL:** http://www.nencki-genomics.org.

## Introduction

Analysis of gene co-regulation requires programmatic access to large amounts of regulatory genomics data, such as the coordinates of genes, chromatin modifications, transcription factor (TF) binding sites and/or motifs. It is often preferred to access such data in a relational database, such as EBI Ensembl database (1). Importantly, this database provides data generated by several other projects, including ENCODE (2), VISTA Enhancer Browser (3) and cisRED (4). However, in Ensembl, the relevant data are spread among several relatively complex schemas, namely, funcgen, compara, and core. Moreover, in the Ensembl database, it is not possible for the user to upload, manage and share own private data or to compute genome-wide intersections between genomic features (overlap on the genomic sequence). Such intersections can easily be computed with external programs, such as BED-tools (5, 6) or ChIPseeqer (7), but analysis of the result in a relational database requires export of the data from the Ensembl database, running the computation, and import of the result into another database.

We developed a database system, named the Nencki Genomics Database (NGD), which for the three species currently represented in Ensembl funcgen (i.e. human, mouse and rat) extends the data and functionality of Ensembl funcgen. To the Ensembl-derived data, we add information on conserved non-coding (putative regulatory) sequences, and on genome-wide occurrences (instances) of transcription factor binding site (TFBS) motifs, from the current versions of two major motif libraries: public Jaspar (8, 9) and (for the most recent NGD version 71_1) also commercial Transfac Professional (Biobase).

**Figure 1.**
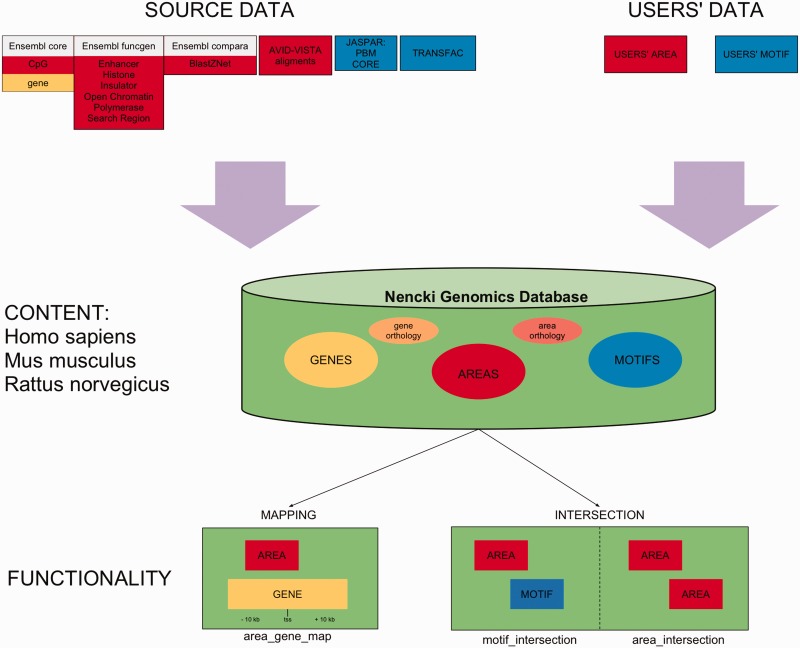
A diagram presenting overview of content and functionality of NGD.

NGD contains public data—derived from Ensembl or provided by us, and data submitted by users, which can be made public by the user. For efficiency reasons, in the database, we separate the instances of TFBS motifs from the remaining regulatory features, which we term areas. In addition to SQL queries, NGD provides procedures for (i) genomic data analysis—area–gene mapping, area–area intersections and area–motif intersections and (ii) data management—addition/removal, managing access rights and making the data public (Figure 1). The results of the intersections are pre-computed for the public data, and similarly the results of area–gene mapping. The amount of added intersection data is significant, with over 6 billions of area–motif intersections (Table 1). NGD is accessible from a mysql client, and its schema is optimized for regulation-related queries.

**Table 1.** Pre-computed data in NGD version 71_1

Database statistics	Human	Mouse	Rat
Number of area data sets	551	69	3
Number of area instances	11994513	2390956	1313917
Number of area intersections	276694188	6277878	57406
Number of motifs instances (Jaspar)	324078012	269409542	207574820
Number of area–motif intersections (Jaspar)	1314579931	237809904	77528929
Number of motifs instances (Transfac)	1229116246	989775972	763762275
Number of area–motif intersections (Transfac)	5255764878	921893795	301210194

The numbers of motif data sets were the same for every species: Jaspar core vertebrate: 146, Jaspar PBM: 208, Transfac: 1476.

A SOAP/WSDL webservice layer provides API to the database procedures, and an additional functionality of graphical visualization of selected NGD content. The webservice can be accessed from a GUI-based client, such as Taverna Workbench. The public areas added by us (excluding the data from Ensembl) and by other users can also be visualized as external DAS tracks in Ensembl Genome Browser. We are currently working to add to NGD a web browser-based interface, as part of a portal integrating genomic and expression data with analysis tools.

## NGD architecture

### Schemas

NGD is versioned, corresponding to the underlying version of Ensembl. Owing to efficiency and access control issues, NGD contains four schemas per each NGD version and species. For example, NGD version 71_1 (Ensembl v.71, local NGD version 1) for the human has four schemas (*71_1_hsap_base_public*, *71_1_hsap_base*, *71_1_hsap_public, 71_1_hsap_users*), of which two are the ‘base’ schema (7*1_1_hsap_base_public, 71_1_hsap_base*) containing tables with the actual data (public and submitted by the users, respectively). The ‘base’ schemas are not directly accessible/visible to the user, who has access to two schemas: ‘public’ and ‘users’ (in this example: *71_1_hsap_public* and *71_1_hsap_users*) containing views to the corresponding tables of the appropriate ‘base’ schema.

### Access control

The access control, built into the ‘users’ schema, operates on a per user and data set basis. For each user and data set, there are two possible access levels: ‘owner’, who can do anything with the data and manage access of other users; and ‘reader’, who can see but cannot modify the data or the access rights.

### Tables/views

**Figure 2.**
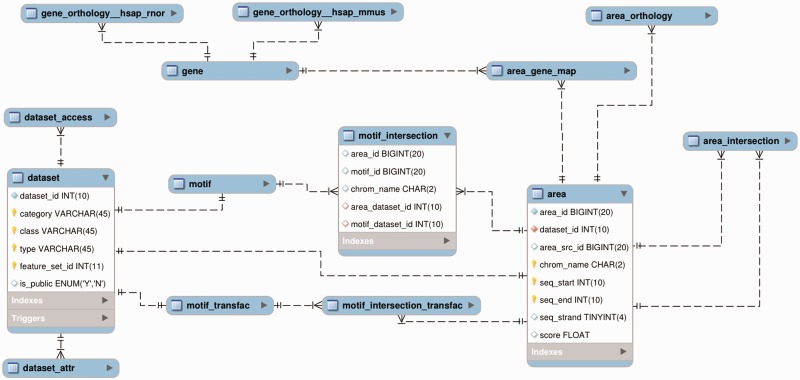
The main tables/views of the NGD ’public‘ and ’users‘ schemas. The table *motif* is nearly identical to the table *area*, with the only difference that its main index column is named *motif_id*, not *area_id*. The table *area_intersection* has the same structure as the table *motif_intersection*, only has different column names *area1_id*, *area2_id*; instead of: *area_id*, *motif_id*.

Similarly to Ensembl, the schema for each NGD version and species contains identical tables/views (Figure 2). The views in the ‘public’ and ‘users’ schema are named the same, but provide access to different sets of data, respectively: all the public data, and all the users-supplied data to which a given user has access. The key to the database is the table *dataset* describing each data set in the database, in terms of its class and type, following Ensembl funcgen. The data sets derived from Ensembl funcgen can be mapped back to it using the funcgen *feature_set_id*. The actual genomic positions of areas and motifs (i.e. motif instances) are contained in the tables *area* and *motif* (for Jaspar and user-added motifs) or *motif_transfac* (for Transfac). The results of mapping areas to genes are stored in the table *area_gene_map*. The area–area intersections reside in the table *area_intersection*, whereas the area–motif interesections reside in the tables *motif_intersection* (Jaspar and user-added motifs) and *motif_intersection_transfac* (for Transfac). The separation of *motif* and *motif_intersection* data between Jaspar and Transfac is for efficiency reason. The mappings of Jaspar TFBS motifs to the transcription factors, as well as other information about data sets, such as a data set name and a cell type of origin for experimental data, are stored as name:value pairs in the table *dataset_attr*, linked to the table *dataset* by *dataset_id*. The Transfac license prohibits us from making public the TF-motif mappings, instead, the table *dataset_attr* contains links to the motifs’ entries on the Transfac web site.

Additionally, the ‘users’ schema contains the so-called input tables, named with the ending ‘*__in_table*’, used by the procedures. The users are allowed to execute SELECT, INSERT, DELETE queries on the input tables, for the rest of the tables only SELECT queries and access through the procedures are permitted. Temporary tables can be created in the *tmp_tables* schema. The temporary tables are session-separated and therefore private to each user.

### Procedures

The ‘public’ schema contains stored procedures for data analysis, which permit genome-wide area–gene mapping, area–area intersections and area–motif intersections. The ‘users’ schema, in addition to the procedures for data analysis, contains procedures for data and access rights management. All the procedures operate at a whole data set level.

The area–gene mapping procedure maps the areas (from the given list of data sets) that intersect (overlap or are contained in) the ±10 kb flank of the transcription start site of every gene to that gene. Different area–area intersection procedures compute intersections between (features from) given pairs of data sets, between all possible pairs of data sets and intersections of one data set with all the other data sets (to which a given user has access). Different area–motif intersection procedures compute intersections between given pairs of area and motif data sets, intersection of a given area data set with all motif data sets, intersection of a given motif data set with all area data sets. The choice of the right intersection procedure is facilitated by the use of webservice (see later in the text).

The procedures for data and access management permit addition/deletion of a data set and (optionally) data set attributes, granting/revoking access and making a data set public. There are mechanisms for maintaining the database integrity, for example deletion of a data set results in removal of all its intersections. The operation of making a data set public is irrevocable and a public data set cannot be deleted.

### Algorithms

The procedures for mapping and intersections use variants of the efficient sweep line algorithm (10). The core algorithm, common to all these procedures, operates as follows:

1. All intervals are placed in the set W sorted by the left end;
2. The sweep (S) is initially empty;
3. The sweep always contains intervals, which are starting before or exact at the x-coordinate of the line (representing the sweep) and which are ending after that x-coordinate.

Algorithm iterative step scenario:

1. Take next interval (K) from W;
2. Delete from S all the intervals that end before K starts;
3. Add K into S and return pair-wise intersection of each pair in S.

All our data analysis procedures were tested, by comparing their results, on a number of data sets, to the results of identical or equivalent (in the case of area–gene mapping) analysis performed with BED-tools (6), with the BED-tools results treated as the gold standard.

### Webservice/clients

A WSDL webservice available at URL http://webservices.nencki-genomics.org/genomic?wsdl provides access to the functionality of the stored NGD procedures from any SOAP client. The webservice functions are augmented in several respects compared to the stored procedures. For example, the webservice function automatically chooses the correct intersection procedure based on the input data. The webservice operations that take a long time provide support for long-running jobs—they return an identifier (id) of the submitted job and send email notifications of job submission and completion. We prepared python clients, taking arguments and options on the command line, which facilitate the use of the webservice. There are separate clients for loading, management, analysis and plotting of the data. We also provide a GUI based interface to the plotting function in the form of a Taverna workflow. These clients can be downloaded from the Webservice section of the online documentation (http://www.nencki-genomics.org/wiki/doku.php?id=tutorial:webservices).

## Example NGD use

The results of the intersection and mapping procedures are pre-computed for the public data (Table 1). Therefore, many questions of biological interest can be answered using SQL queries. The versatility of NGD stems from the possibility of performing arbitrary SQL queries on the intersection and mapping data. For example, to identify all the instances of the Jaspar motifs in the rat AVID–VISTA regions and to map them to the genes, the user could execute the following commands at the mysql command prompt:

USE 71_1_rnor_public;

SET @ds_id= (SELECT dataset_id from dataset WHERE class='AVID-VISTA');

CREATE TEMPORARY TABLE tmp_tables.av_jaspar SELECT area_id, motif_id, motif_dataset_id FROM motif_intersection WHERE area_dataset_id=@ds_id;

ALTER TABLE tmp_tables.av_jaspar ADD INDEX(area_id);

CREATE TEMPORARY TABLE tmp_tables.gene_av_jaspar SELECT b.gene_id, a.* FROM tmp_tables.av_jaspar AS a JOIN area_gene_map AS b USING(area_id);

For all operations on the public data, a user does not need the database procedures, as their results on all these data have been pre-computed and are stored in the database. The procedures become necessary when the user wants to analyze own data in the context of the public data or data shared by another user.

Upload of user-supplied area data is possible using the database procedure *area__reload__proc* or the webservice function calling this procedure, and similarly for motifs and data set attributes. We recommend upload via the webservice, which is both easier (no need to directly fill the input tables) and more convenient (support for long jobs with email notification). The use of the webservice is facilitated by the provided python clients accepting command line arguments and options. Notably, called with the -h or –help option, each client returns a full description of all its arguments and options. The uploaded data must be in one of two formats: the BED format, described at the UCSC website (http://genome.ucsc.edu/FAQ/FAQformat) or the NGD format, described in the documentation online (http://www.nencki-genomics.org/wiki/doku.php?id=tutorial:webservices). To upload an example area data set using the python client, the user invokes it from the command line, providing own database credentials, database version, species, path to the data file and the data set description in terms of its type and class:

client__load.py -u <user> -p <passwd> -v 71_1 -s rnor -f/path_to_data/area_to_load.tsv -t 'test area' -c 'test class'

Once the user-supplied data are in the database, they can be analyzed and managed using the stored database procedures. As an example of calling a stored procedure, the user could execute the following commands to compute the intersections between two pairs of area data sets, with the result stored in the table a*rea_intersection*:

INSERT INTO area_intersection__dataset_map__in_table(dataset1_id,dataset2_id) VALUES (1,2),(3,^4^);

CALL area_intersection__map__proc;

The use of the remaining procedures is similar. Basically, the user needs to fill in an appropriate input table(s) and then call the procedure. There are mechanisms preventing duplication if a user attempts to compute a result already in the database. All the database procedures can be accessed via the webservice, notably also using a GUI client, such as Taverna Workbench.

Additionally, the webservice-only function PlotGenomic plots a graphical representation of selected NGD content in the ±10 kb flank of the transcription start site of a chosen gene and also returns this content as tab-separated files. More precisely, this function returns instances of selected area types, and instances of selected motif types that intersect any of the returned area instances. This function can be conveniently accessed using the command line client, also by the public (i.e. unregistred) user. For example, to plot all area types and selected motif types intersecting these areas, in the ±10 kb flank of the human BDNF, the user may execute the following command at the comand line:

client__plot_genomic.py -u public -s hsap -l jaspar -g BDNF -m CREB1,CTCF,REST\

-a Yy1,Nrsf,CTCF,DNase1

**Figure 3.**
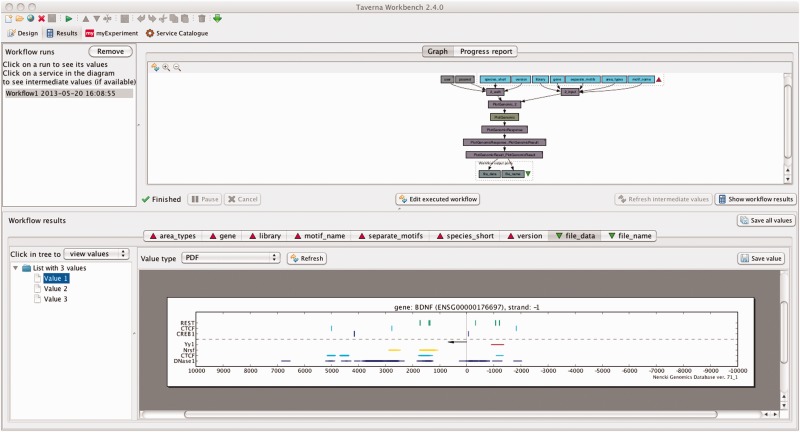
Visualization of selected NGD content in the ±10 kb flank of a gene from Taverna Workbench. The plotting function returns the motifs of the chosen type that intersect any of the shown areas.

Importantly, all the functions of our webservice can also be called using the GUI interface of Taverna Workbench. Figure 3 shows a screen-shot of the same plot generated from Taverna Workbench.

## Methods

### Import from Ensembl

The main source of the data in our database for the human and mouse is Ensembl funcgen, from which we import all the features in the classes: Enhancer, Histone, Insulator, Transcription Factor, Open Chromatin, Polymerase, Search Region. The names of funcgen feature_sets (containing the ENCODE names) can be found in the table *dataset_attr*. The processing of the ENCODE data is described by the Ensembl team under this link http://ftp.ebi.ac.uk/pub/databases/ensembl/encode/integration_data_jan2011/hg19/uniformTfbs.html. The ‘Search Region’ class contains the promoters’ predictions—imported into Ensembl funcgen from the cisRED database (http://www.cisred.org/) (4). To these data, we add information about genes and CpG islands from Ensembl core, and about pairwise genome alignments (BlastZNet/LastZNet) (11) from Ensembl compara. The LastZNet features are additionally filtered—we retain only those with the length between 100 and 5000 base pairs. For each Ensembl build and species, we use only one coordinate system—the most current chromosomal one.

### AVID–VISTA alignments

The ±10 kb genomic flanks of transcription start sites of orthologous genes (Ensembl: ortholog_one2one, apparent_ortholog_one2one) are aligned with avid (12). The alignment is processed with the program VISTA (13) using the default parameters (minimum length: 100 nt, minimum identity: 75%, excluded exons). The chromosomal coordinates of the resulting conserved non-coding regions are imported into the database, filtering away possible duplications.

### Genome-wide TFBS motif finding

For finding TFBS motifs in the genomic sequences downloaded from Ensembl, we use the command line version of the program matrix-scan (quick) (14), using the pre-computed first order (2 nt.) Markov-chain background models for each species, based on both strands of the upstream non-coding regions of the genome, e.g. 2nt_upstream-noorf_Homo_sapiens_EnsEMBL-ovlp-2str for human. The motif scores are converted to *P*-values, based on the distributions of scores computed for each motif using the program matrix-distrib. A uniform *P*-value threshold of *P* < 0.0001 is used to call an instance of a particular motif at a given genomic position. We used motifs from the current (12 October 2009) version of Jaspar, including Jaspar core vertebrate (8) and Jaspar PBM (9). For the most recent NGD version 71_1, we also used all the vertebrate motifs from the current version (Spring 2013.1) of Transfac Professional (Biobase).

## Discussion

The solutions alternative to NGD include

- reference genomic databases and genome browsers: Ensembl (1), UCSC (15)
- regulatory genomics databases, including VISTA Enhancer Browser (3), cisRED (4), EELWeb (16) MAPPER2 (17), D-light on promoters (18)
- programs for analysis of motifs content of regulatory regions, including Toucan 2 (19), cREMaG (20), oPOSSUM-3 (21)
- programs for analysis of next-generation sequencing data, including BED Tools (5, 6) and ChIPseeqer (7).

NGD presents several amendments over the existing solutions listed earlier in the text. Precomputed instances of Jaspar motifs in Ensembl funcgen are provided only within the ChIP-seq regions and are limited to the motifs for the TFs represented in the ChIP-seq data, whereas in NGD, all Jaspar and Transfac motifs are provided in the whole genome (Table 1). Similarly, the aforementioned regulatory genomics databases contain TFBS motif instances only in predicted regulatory regions, not in the whole genome.

The intersection functionality offered by UCSC is limited, as the UCSC Table Browser intersection procedure does not return the full information about the intersecting features (as does NGD). Instead, it returns either (i) the full information on the features from one table, but no information on the features from the other table or (ii) nucleotide positions of the regions in the intersection, but with no reference to the original tables. Moreover, UCSC intersections interface is not designed for running extensive (e.g. all pairwise) intersections. The aforementioned programs for analysis of motif content of regulatory regions do not permit intersecting areas.

BED Tools, available as a standalone program and a python library, offers rich functionality for intersections, comparable with that of NGD. ChIPseeqer provides functionality of area intersection, gene mapping and finding of known public TFBS motifs. The main difference between our system and the last two programs is the fact that in NGD, the result of any procedure is stored back in the database and is available to all users with access rights to the underlying data sets, thus incrementally increasing the body of pre-computed data. In particular, the results for a data sets that are made public also become public.

## Funding

This work was supported by European Regional Development Fund, under the Operational Programme Innovative Economy [POIG 02.03.00-00-003/09]. Funding for open access charge: European Regional Development Fund.

*Conflict of interest*. None declared.

## References

[1] Flicek P, Ahmed I, Amode MR (2013). Ensembl 2013. Nucleic Acids Res..

[2] Dunham I, Kundaje A, Aldred SF (2012). An integrated encyclopedia of DNA elements in the human genome. Nature.

[3] Visel A, Minovitsky S, Dubchak I (2007). VISTA enhancer browser–a database of tissue-specific human enhancers. Nucleic Acids Res..

[4] Robertson G, Bilenky M, Lin K (2006). cisRED: a database system for genome-scale computational discovery of regulatory elements. Nucleic Acids Res..

[5] Dale RK, Pedersen BS, Quinlan AR (2011). Pybedtools: a flexible Python library for manipulating genomic datasets and annotations. Bioinformatics (Oxford, England).

[6] Quinlan AR, Hall IM (2010). BEDTools: a flexible suite of utilities for comparing genomic features. Bioinformatics (Oxford, England).

[7] Giannopoulou EG, Elemento O (2011). An integrated ChIP-seq analysis platform with customizable workflows. BMC Bioinformatics.

[8] Bryne JC, Valen E, Tang M-HE (2008). JASPAR, the open access database of transcription factor-binding profiles: new content and tools in the 2008 update. Nucleic Acids Res..

[9] Badis G, Berger MF, Philippakis AA (2009). Diversity and complexity in DNA recognition by transcription factors. Science (New York, NY).

[10] De Berg M, Cheong O, van Kreveld M (2008). Computational Geometry Algorithms and Applications.

[11] Kent WJ, Baertsch R, Hinrichs A (2003). Evolution’s cauldron: duplication, deletion, and rearrangement in the mouse and human genomes. Proc. Natl Acad. Sci. USA.

[12] Bray N, Dubchak I, Pachter L (2003). AVID: a global alignment program. Genome Res..

[13] Mayor C, Brudno M, Schwartz JR (2000). VISTA: visualizing global DNA sequence alignments of arbitrary length. Bioinformatics (Oxford, England).

[14] Turatsinze J-V, Thomas-Chollier M, Defrance M (2008). Using RSAT to scan genome sequences for transcription factor binding sites and cis-regulatory modules. Nat. Protoc..

[15] Meyer LR, Zweig AS, Hinrichs AS (2013). The UCSC genome browser database: extensions and updates 2013. Nucleic Acids Res..

[16] Hallikas O, Palin K, Sinjushina N (2006). Genome-wide prediction of mammalian enhancers based on analysis of transcription-factor binding affinity. Cell.

[17] Riva A (2012). The MAPPER2 database: a multi-genome catalog of putative transcription factor binding sites. Nucleic Acids Res..

[18] Laimer J, Zuzan CJ, Ehrenberger T (2013). D-Light on promoters: a client-server system for the analysis and visualization of cis-regulatory elements. BMC Bioinformatics.

[19] Aerts S, Van Loo P, Thijs G (2005). TOUCAN 2: the all-inclusive open source workbench for regulatory sequence analysis. Nucleic Acids Res..

[20] Piechota M, Korostynski M, Przewlocki R (2010). Identification of cis-regulatory elements in the mammalian genome: the cREMaG database. PloS One.

[21] Kwon AT, Arenillas DJ, Worsley Hunt R (2012). oPOSSUM-3: advanced analysis of regulatory motif over-representation across genes or ChIP-Seq datasets. G3 (Bethesda, Md.).

